# What helps people with mental illness to function in daily life? Findings from a Delphi study

**DOI:** 10.1186/s12888-026-08215-6

**Published:** 2026-06-06

**Authors:** Walton Wider, Amrita Deb, Chunwen Yang, Nicholas Tze Ping Pang, Changhe Wu, Hao Wu, Alex S. Borromeo, Danni Wu

**Affiliations:** 1 Faculty of Business and Communications, INTI International University, Nilai, Malaysia; 2 Department of Liberal Arts, Indian Institute of Technology Hyderabad, Sangareddy, India; 3 School of Advanced Translation and Interpretation, Dalian University of Foreign Languages, Dalian, China; 4 College of Foreign Languages, Tarim University, Alar, Xinjiang China; 5 Faculty of Medicine and Health Sciences, Universiti Malaysia Sabah, Kota Kinabalu, Malaysia; 6 College of Nursing, Bulacan State University, Malolos, Philippines; 7 Faculty of Liberal Arts, Shinawatra University, Pathum Thani, Thailand

**Keywords:** Functional recovery, Mental illness, Delphi study, Expert consensus, Recovery-oriented practice, Psychosocial rehabilitation, Digital mental health

## Abstract

**Background:**

Functional impairment remains a central yet insufficiently addressed challenge in mental health care, as many systems continue to prioritise symptom containment over sustained participation in daily life. This study aimed to identify and prioritise strategies that support optimal functioning among individuals living with mental illness by integrating psychosocial, community-based, and digital approaches within recovery-oriented and biopsychosocial frameworks.

**Methods:**

A modified Delphi design was employed with 18 experts from clinical, counselling, and academic settings. In Round 1, participants generated strategy statements that were synthesised through thematic analysis, supported by AI-assisted coding and subsequent human review, into eight dimensions of functional support. In Round 2, 16 experts ranked these dimensions by perceived importance, and Kendall’s coefficient of concordance was used to assess consensus.

**Results:**

The final framework comprised: Medical and Professional Support; Social and Family Support; Physical Health and Wellness; Emotional and Cognitive Coping; Mindfulness and Spiritual Practice; Structured Daily Living; Self-Directed Support and Autonomy; and Functional Adaptation and Masking. Experts prioritised formal clinical care, relational support, and physical health as foundational, with everyday self-regulation and structured routines occupying mid-level importance, and masking-based or high-effort compensatory strategies ranked lowest. Kendall’s W indicated statistically significant but modest agreement, consistent with the expert’s heterogeneity.

**Conclusions:**

The findings recast functional recovery as an emergent property of treatment access, social ecology, lifestyle stability, and self-regulation, while highlighting the ethical limits of placing responsibility for functioning primarily on individuals. The framework offers a practice-oriented scaffold for clinicians, service planners, and policymakers seeking to reorient mental health systems toward cultivating meaningful, sustainable everyday living.

**Clinical trial number:**

Not applicable.

## Introduction

Efforts to enhance the lives of individuals living with mental illness continue to encounter a persistent structural dilemma. Mental illness refers to conditions that affect cognition, emotion, and behaviour, such as schizophrenia, depression, and autism [62]. Although clinical knowledge and treatment modalities have advanced considerably, many individuals still experience significant disruptions in daily functioning and work ability, as noted by researchers such as Tanner et al., [92]. Difficulties in motivation, social interaction, illness self-management, and independent role performance remain common, and these challenges shape long-term well-being and prospects for social inclusion [37, 51]. Functioning in the context of health and mental health conditions is commonly assessed by instruments such as the World Health Organisation Disability Assessment Schedule 2.0 (WHODAS 2.0) [85]. This instrument, for instance, assesses functioning on six domains: cognition, mobility, self-care, getting along, life activities, and participation. In studying functioning and symptom severity, Backenstrass et al., [7] reported that those with subthreshold depression were significantly affected in daily activities and functioning in contrast to nondepressive primary care patients. These functional impairments are central rather than peripheral, yet many mental-health systems remain oriented toward symptom containment instead of supporting the broader capacities required for meaningful and self-directed living. This misalignment has created a sustained gap between the services that are typically provided and the forms of support individuals need to function effectively in everyday life.

Although a wide range of psychosocial, digital, community-based, and peer-support interventions is available, the evidence base remains uneven and fragmented across disciplinary boundaries [11, 84, 96]. Digital therapeutics have shown promise in promoting accessibility and engagement, yet their integration into established rehabilitation pathways continues to raise methodological and implementation concerns [32, 68, 79]. Likewise, community-based and peer-led models contribute to empowerment and participation, though outcomes vary widely depending on contextual resources, social capital, and institutional readiness [60, 74, 88].

Conceptual research reinforces the idea that functional improvement cannot be understood solely in terms of symptom relief. The World Health Organisation’s (WHO) International Classification of Functioning, Disability and Health provides an understanding of health beyond clinical diagnosis, emphasising components such as functioning and disability [103]. In addition, the [103] presents environmental factors as external influences on functioning, covering the following components: products and technology, natural environment and human-made changes, support and relationships, attitudes, services, systems, and policies. These components present a holistic framework for recognising functioning and health. Recovery encompasses personal growth, clinical stability, and the practical capacities required to fulfil roles and responsibilities, with each dimension shaping and reinforcing the others [22, 83]. Optimal functioning is supported by illness self-management, psychosocial rehabilitation, engagement in purposeful activity, and participation in valued social roles [12, 90]. The Recovery Model, the Biopsychosocial Model, and behavioural self-management frameworks share the view that recovery is relational and contextual, emerging within environments that foster autonomy, competence, and connectedness [23, 89].

Despite the breadth of available interventions, a critical gap persists. The field lacks a consolidated, theory-driven, empirically informed framework that identifies the strategies most essential for promoting functional outcomes. The absence of such integration results in varied practices, limited transferability across settings, and inequitable access to recovery-oriented care. These limitations restrict opportunities for long-term recovery and reinforce patterns of social exclusion [47].

The present study seeks to address this gap by synthesising and evaluating strategies that support optimal functioning in daily life. These strategies may include conscious cognitive and/or behavioural efforts individuals use to manage their symptoms and other ongoing stressors. Conceptually, these strategies align with established constructs in the literature, including coping strategies and self-management processes, which emphasise individuals’ active role in regulating emotional, cognitive, and behavioural responses to sustain functioning [5, 46, 87]. By integrating psychosocial, community-based, and digital approaches, the study aims to establish a coherent conceptual foundation that guides practice, informs policy directions, and shapes future research, reflecting evidence that multicomponent and technology-enabled interventions can enhance self-management, well-being, and functional outcomes [2, 15, 59, 86]. This work aligns with a growing commitment within the field to reframe mental-health support as the cultivation of human capacities, agency, and participation, emphasising self-efficacy, empowerment, and resilience, rather than reducing mental health to pathology alone [19, 87].

## Literature review

Research on mental-health functioning has shifted considerably in recent decades. Earlier approaches centred on symptom management, whereas contemporary perspectives emphasise recovery, autonomy, and sustained participation in daily activities. This shift is evident in the growing adoption of recovery-oriented, community-based rehabilitation frameworks that prioritise psychosocial functioning and quality of life as intervention goals [8, 25]. Functional impairment is now widely regarded as a defining feature of mental illness with implications for independence, identity, and social belonging.

### Conceptual foundations of functional recovery

The literature consistently situates functional recovery within a broader triad that includes personal and clinical domains. Personal recovery involves hope, empowerment, and identity reconstruction. Clinical recovery focuses on symptom stability, whereas functional recovery concerns the capacity to perform roles, maintain routines, and meet social expectations [22, 83]. These domains operate interdependently. Gains in functioning can strengthen personal agency, while personal empowerment improves treatment engagement. Interventions that integrate illness self-management, psychosocial rehabilitation, and structured activity demonstrate the strongest and most holistic outcomes [12, 84].

The objective of the present study was to identify and refine the strategies individuals most commonly use to achieve optimal functioning. Considering that the participants were working with clients exhibiting varied levels of symptom severity, recovery and adaptation were understood to overlap. In this context, the terms recovery and adaptation refer to the range of outcomes associated with clinical recovery, personal recovery, and adaptation to living with symptoms and other co-occurring conditions. Accordingly, the review covered interventions that trained individuals to apply strategies for recovery or adaptation, that is, reduction in symptoms, as well as adapting to living well with symptoms. Additionally, literature related to the mode of application (e.g., hybrid and digital) and functional outcomes is reported below.

### Psychosocial and community-based interventions

Community-based and psychosocial interventions continue to hold a prominent place in functional rehabilitation. Supported housing initiatives are associated with improvements in quality of life and social engagement when they include personalised supports that reflect individual goals [90]. Psychosocial rehabilitation programs similarly enhance self-management of illness while fostering both functional and personal recovery [83]. These models draw on behavioural and cognitive principles that emphasise skill development and reinforcement through daily environments.

Despite their promise, psychosocial models face practical barriers. Organisational readiness, inconsistent resource availability, and variation in staff competencies influence scalability and reliability of outcomes [90]. Even so, research across diverse settings supports the conclusion that functioning improves most when supports are tailored, flexible, and embedded in community contexts that carry personal meaning for the individual.

### Digital and hybrid interventions

Digital technologies have expanded rapidly in mental health care and now offer scalable mechanisms for monitoring symptoms, supporting self-management, and complementing face-to-face interventions. Studies increasingly document improvements in functioning, quality of life, and adherence among users of digital and hybrid psychosocial programs [4]. Digital interventions for first-episode psychosis further demonstrate benefits in accessibility and functional outcomes, although engagement and technology acceptance remain important challenges [98]. Digital therapeutics for schizophrenia spectrum disorders show potential yet vary in effectiveness depending on the degree of personalisation and integration with human support [32]. Peer involvement enhances the use of digital tools by strengthening motivation and accountability [30]. These findings highlight the value of hybrid models that bring together the efficiency of technology with the relational depth of human support.

### Coping, skills training, and self-management

Coping strategies and everyday skills contribute significantly to functional outcomes. Preventive coping supports mood regulation and adaptive functioning [104]. Skills-based rehabilitation approaches that emphasise autonomy, community integration, and social competence have been reported to improve social and vocational functioning [43, 100]. Interventions developed through shared decision-making often lead to stronger functional gains that align with personal preferences and goals [77]. Research further suggests that diverse coping repertoires are protective and that coping flexibility is critical for adapting to changing contexts [76]. Within the present study, the term “strategy” is used as an umbrella construct encompassing these coping and self-management processes, reflecting the diverse ways individuals actively engage in maintaining functional recovery.

### Social support and relational recovery

Social support is one of the most consistent predictors of functional improvement. Supportive relationships promote stability, enhance role functioning, and shape long-term recovery trajectories [24, 29]. These findings correspond with relational models of recovery that view functioning as a product of interactions between individual capacities and the broader social ecology. Peer relationships, family involvement, and community networks reinforce motivation and reduce the likelihood of relapse, thereby creating environments where functional gains can be sustained.

The literature presents a complex range of psychological, social, and environmental influences on functional outcomes. Overall, psychosocial, community-based, and digital approaches aim to build, implement, and reinforce behaviours that contribute to optimal functioning. While these approaches are often studied separately, in practice, they function in interlocking ways rather than in isolation. For instance, cognitive and behavioural techniques used in psychosocial and community-based approaches may be applied together to identify supportive systems and foster help-seeking when in distress. Digital approaches, on the other hand, can be used to schedule and remind participants to practice these techniques. Although many strategies have demonstrated value, the diversity of approaches and the variability in implementation highlight the need for structured expert consensus. This need provides a clear rationale for employing a modified Delphi design in the present study, enabling experts to identify and refine the strategies most critical to supporting optimal functioning.

## Methods

### Research design

This study used a modified Delphi design to identify and prioritise strategies that promote optimal functioning among individuals with mental illness. The Delphi technique is a structured, multi-round process that relies on expert judgment to build consensus on topics where evidence may be incomplete or dispersed [52, 102]. This approach was well-suited to the present study because optimal functioning spans multiple domains that are not easily captured by a single cross-sectional survey or interview. These domains include symptom management, role performance, social participation, self-care, and quality of life, all of which play interdependent roles in functional recovery [39, 53]. Therefore, systematic, structured expert judgments were expected to cover a broader range of information than traditional approaches, such as qualitative interviews or surveys. While qualitative approaches may have helped identify unique responses, they would not have allowed the expert panel to build consensus through progressive refinement of their responses. On the other hand, the Delphi design enabled experts to provide a broad range of insights and to refine their judgments across iterative rounds, ensuring that the final set of strategies reflected shared professional understanding and feasibility within real-world mental health settings.

Consistent with the study’s objective of prioritisation, the Delphi process was designed to generate a relative ordering of strategies, with consensus assessed by the degree of agreement among expert rankings rather than by independent item ratings.

The Delphi procedure followed best-practice guidelines for consensus development [102]. Participation was anonymous, questionnaires were administered online, and controlled feedback was provided between rounds. Anonymity reduced the risk of group pressure or the undue influence of more senior contributors, and the iterative structure allowed experts to reconsider earlier responses in light of aggregated group feedback [65]. Quantitative indicators of agreement and qualitative comments were used together to refine strategy statements across rounds [99]. In this context, consensus was operationalised as the degree of agreement in ranking patterns across experts, rather than convergence in independent ratings, thereby allowing the study to capture both shared priorities and variability in expert perspectives. These methodological features supported a rigorous, transparent, and systematic approach to consensus building.

This study adhered to the Guidance on Conducting and Reporting Delphi Studies (CREDES). The research design, expert selection procedures, definition of consensus, iterative feedback process, and reporting of results were structured in accordance with CREDES recommendations to enhance methodological transparency, rigour, and reproducibility.

### Participants

The study recruited experts who work with or conduct research on individuals with mental illness across clinical, counselling, and higher-education contexts, with clients varying in symptom severity. Purposive sampling ensured that all participants possessed substantial expertise in mental health and psychosocial functioning [38]. Experts were selected based on academic qualifications in psychology, psychiatry, counselling, or related fields; professional roles such as clinicians or academic staff; and demonstrable experience in assessment, treatment, or mental health research [70]. All participants held at least a bachelor’s degree, and most possessed postgraduate qualifications with several years of professional experience.

Table 1 presents a comprehensive profile of the Round 1 expert panel. A total of 25 experts were invited to participate; 18 completed the first round, yielding a response rate of 72 per cent. The 18 experts who participated in the first round fall within the recommended Delphi panel size of 15 to 30 participants, which is considered sufficient to ensure diversity of perspectives while maintaining manageability across iterative rounds [94]. Consistent with research on qualitative saturation literature, this sample size is adequate to identify core conceptual patterns, as additional participants typically refine rather than substantially alter the overall thematic structure [45]. The panel comprised experts primarily based in Malaysia, with additional representation from the United Kingdom, Indonesia, and India, thereby ensuring regional relevance while incorporating international perspectives.

Table 1 provides a detailed profile of the experts, demonstrating a balanced, diverse group of experts across demographic and professional dimensions. The experts ranged in age from their early 30s to late 40s, representing primarily mid-career professionals who possess both substantial field experience and contemporary knowledge of mental health practice. The gender distribution was relatively balanced, contributing to a broader representation of perspectives.

All experts hold at least a bachelor’s degree, with the majority having completed master’s degrees and several holding doctoral qualifications. This reflects a high level of academic preparation aligned with the expectations of Delphi experts that require advanced conceptual and practical expertise. Professional experience ranged from less than 10 to over 30 years, with most participants having 11–20 years, indicating deep familiarity with clinical, counselling, and academic mental health contexts.

The expert also represented a wide range of professional roles, including associate professors, clinical psychologists, psychiatrists, counsellors, lecturers, and senior administrators such as deans and vice deans. This interdisciplinary composition strengthened the methodological rigour of the Delphi process by incorporating insights from multiple subfields of mental health, ensuring that varied yet complementary forms informed the identified factors and subsequent consensus of expertise.

**Table 1:** List of experts who participated in the first round of the Delphi study (*N* = 18)

No.	Gender	Age	Academic Qualification	Working Experience	Position
E1	Male	39	Master	11–20 years	Associate Professor
E2	Female	35	PhD	11–20 years	Consultant Clinical Psychologist
E3	Male	35	Bachelor	11–20 years	Lecturer
E4	Female	36	Master	11–20 years	Head of Student Counselling Unit
E5	Female	47	PhD	21–30 years	Dean / Professor
E6	Female	43	Master	11–20 years	Clinical Psychiatrist
E7	Female	36	Master	Less than 10 years	Senior Student Counsellor
E8	Female	49	PhD	21–30 years	Trainee in Child and Adolescent Psychiatry
E9	Male	34	Master	Less than 10 years	Clinical Psychiatrist
E10	Male	40	Master	11–20 years	Consultant psychiatrist
E11	Female	35	Master	11–20 years	PhD Student
E12	Female	38	PhD	11–20 years	Senior lecturer
E13	Male	40	PhD	11–20 years	Vice Dean
E14	Male	33	Master	Less than 10 years	Lecturer
E15	Male	35	Master	11–20 years	Psychiatrist
E16	Male	38	Master	11–20 years	Dietitian
E17	Female	39	Master	11–20 years	Clinical psychologist
E18	Male	34	Master	Less than 10 years	Lecturer

### Procedures

A two-round modified Delphi process was conducted between June 15 and June 30, 2025. The study adopted a streamlined online format to reduce participant burden while maintaining methodological rigour. This format is consistent with recent applications of the Delphi technique in the social sciences, which emphasise efficiency, anonymity, and controlled feedback [13].

In Round 1, experts were invited to generate strategies they considered important for promoting optimal functioning among individuals with mental illness. They were asked to describe each strategy, specify its context of use, and explain its significance [93]. In this study, the term “strategy” was operationalised broadly to capture coping, self-management, and adaptive behavioural practices identified in prior literature. To guide reflection without constraining responses, examples of domains drawn from prior research on recovery and psychosocial functioning were provided, including symptom management, social participation, daily living skills, emotional regulation, and vocational functioning. Experts were encouraged to introduce any additional ideas beyond those represented in the literature. All responses were anonymised and subjected to inductive thematic analysis, through which overlapping ideas were consolidated into domains and coherent strategy statements.

In Round 2, experts were asked to rank the identified dimensions from most to least important. A forced-ranking approach was adopted rather than separate rating scales (e.g., importance and relevance) to elicit clear prioritisation and to reduce central-tendency bias. Ranking requires participants to make trade-offs among options, thereby producing a more discriminative hierarchy of priorities that aligns with the study’s objective of identifying the relative importance of strategies rather than evaluating them independently.

### Data analysis

Qualitative data from Round 1 were analysed using thematic analysis, following Braun and Clarke’s [14] framework and integrating the structured, AI-assisted analytic approach proposed by Naeem et al., [69]. This combined method was well-suited to the present study, as expert responses addressed multidimensional aspects of recovery and functioning that required both conceptual sensitivity and systematic organisation.

Analysis began with familiarisation, during which all responses were read repeatedly to identify early patterns. Consistent with Naeem et al.,’s [69] systematic thematic analysis procedure, ChatGPT was provided with structured prompts that operationalised each stage of analysis. These prompts explicitly embedded the research aim, research questions, theoretical framework, methodological approach, and contextual information, ensuring that the AI system was fully oriented to the analytical task prior to coding. In line with Naeem et al., [69], the prompts were step-specific and instruction-driven. For example, during the familiarisation stage, prompts required the system to review the research context and confirm its understanding before proceeding. Subsequent prompts guided the extraction of salient excerpts, the identification of high-information keywords using structured criteria (e.g., relevance and representativeness), the generation of codes through abductive reasoning, and the clustering of codes into themes grounded in both data patterns and theoretical constructs. This structured prompting ensured that each analytical output was explicitly aligned with the study’s conceptual and methodological foundations rather than generated in isolation.

The system supported the identification of salient excerpts and high-information keywords, functioning as an auxiliary heuristic tool rather than an independent coder. Importantly, all AI-assisted outputs were iteratively reviewed, refined, and validated by the research team, with final decisions on coding and theme development made through human interpretation to ensure analytical rigour and contextual accuracy. This approach aligns with methodological guidance emphasising that AI should support, rather than replace, researcher-led interpretation in qualitative analysis.

Inductive coding proceeded through clustering similar concepts into descriptive codes, followed by iterative movement between data-driven insights and theoretical constructs, a process consistent with abductive reasoning in qualitative research [95]. Codes were refined into candidate themes representing broader strategic domains for enhancing functional recovery. Themes were evaluated for internal coherence, conceptual clarity, and relevance to the study’s aims, yielding a set of domains that accurately reflected shared expert perspectives.

Quantitative data from Round 2 were analysed using Kendall’s coefficient of concordance (W) to evaluate agreement across experts, with significance set at *p* <.05 in accordance with recommended Delphi procedures [94]. Higher W values indicated stronger consensus. As the obtained W met the pre-specified threshold, additional rounds were not required.

### Ethics

This study received approval from the Scientific and Ethical Review Committee. Prior to taking part, all experts were informed about the aims of the research, the measures taken to protect confidentiality, and their freedom to withdraw at any stage. Consent was collected via a digital form embedded in the questionnaire, in which participants confirmed their voluntary participation, agreed to the use of anonymised data, and acknowledged the study conditions. No personally identifiable information is reported in the Results section.

## Results

### First round of the delphi study

The first round of the Delphi study generated 40 strategy statements, which were synthesised into eight thematic dimensions representing the spectrum of strategies that support optimal functioning among individuals living with mental illness (Table 2). It is important to note here that some individual strategies were observed to cut across categories; for example, the use of online self-help tools may be associated with both Structured Daily Living and Self-Directed Support and Autonomy. Both the biopsychosocial and recovery-oriented frameworks jointly informed the coding and interpretation of the reported strategies, with some overlap. While the biopsychosocial model guided us to include aspects of physical and emotional health and mindfulness, such as medication adherence, the recovery-oriented framework led us to emphasise strategies related to Structured Daily Living, Self-Directed Support, and Autonomy, such as independent learning. Many strategies reflected components from both frameworks.

The first dimension, Medical and Professional Support (MPS), encompasses strategies involving consistent engagement with formal mental health services. These include medication adherence, participation in psychotherapy, regular consultations with mental health professionals, and involvement in structured rehabilitation programmes. Supported employment and case management also emerged as salient components, underscoring the importance of multidisciplinary coordination in linking clinical care with functional and occupational outcomes [40].

The second dimension, Structured Daily Living (SDL), reflects strategies that promote routine, predictability, and behavioural organisation. Activity scheduling, maintaining regular work routines, journaling and self-reflection, and the use of digital self-help tools to support time management illustrate how structured environments facilitate stability, continuity, and self-regulation [28].

A second cluster of dimensions emphasises internal regulation, health-promoting behaviours, and meaning-making. Mindfulness and Spiritual Practice (MSP) includes meditation, breathwork, mindfulness exercises, religious or spiritual engagement, gratitude practices, and self-compassion. These strategies highlight the importance of contemplative practices in fostering emotional balance, resilience, and existential grounding [16].

Physical Health and Wellness (PHW) comprises lifestyle-oriented strategies such as regular physical exercise, balanced nutrition, sleep hygiene, avoidance of substance use, and management of comorbid health conditions. These findings reinforce the bidirectional relationship between physical and psychological well-being [35].

Complementing these, Social and Family Support (SFS) captures strategies embedded in interpersonal and relational contexts, such as living with supportive family members, joining support groups, maintaining social ties, cultivating positive workplace relationships, and participating in peer-based networks. These dimensions illustrate that optimal functioning is shaped by the interaction of individual coping capacities and relational environments [44].

The final set of dimensions centres on autonomy, adaptive coping, and the navigation of environmental demands. Emotional and Cognitive Coping (ECC) includes emotional regulation skills, balanced use of short-term distraction, goal setting and prioritisation, acceptance and cognitive flexibility, as well as conflict resolution and boundary-setting skills. Self-Directed Support and Autonomy (SDSA) captures proactive personal agency through the use of self-help resources, online tools, independent learning, behaviour change efforts, and timely help-seeking. These strategies reflect the individual’s active role in sustaining daily functioning and recovery [44, 58].

The final dimension, Functional Adaptation and Masking (FAM), encompasses adaptation strategies such as overcompensation or “apparent competence,” maintaining high productivity, modifying environments or roles, and concealing distress in social or professional contexts. Although these strategies may temporarily support role performance, they also highlight the invisible labour individuals undertake to meet external expectations [20, 40].

**Table 2:** Thematic dimensions of strategies for optimal functioning among individuals with mental illness

No	Dimensions	Themes
1	Medical and Professional Support	• Medication adherence • Participation in psychotherapy (CBT, counseling, emotional regulation therapy) • Follow-ups with mental health professionals • Engagement in rehabilitation programs (e.g., ADL, vocational training) • Supported employment and case management
2	Structured Daily Living	• Activity scheduling (morning/evening routines) • Journaling and reflective practices • Maintenance of work routines • Use of digital self-help tools (apps, online programs) • Time management practices
3	Mindfulness and Spiritual Practice	• Meditation and breathwork • Mindfulness techniques • Religious or spiritual activities • Gratitude practice • Self-compassion and acceptance
4	Physical Health and Wellness	• Regular physical activity (e.g., walking, exercise) • Balanced nutrition • Sleep hygiene • Avoidance of substance use • Attending to comorbid physical health needs
5	Social and Family Support	• Living with family or supportive significant others • Participation in support groups • Maintaining interpersonal connections • Positive workplace relationships • Peer mentoring or shared lived experiences
6	Emotional and Cognitive Coping	• Emotional regulation skills (distress tolerance, awareness of emotional states) • Short-term distraction (e.g., games, work, scrolling) • Goal setting and prioritization • Acceptance and cognitive flexibility • Conflict resolution and boundary-setting skills
7	Self-Directed Support and Autonomy	• Use of self-help resources • Online tools and educational materials • Self-efficacy and motivation • Independent learning and behavior modification • Seeking help when necessary
8	Functional Adaptation and Masking	• Overcompensation and “apparent competence” • High productivity to conceal difficulties • Environmental adjustments (e.g., change of setting/activity) • Role restructuring (e.g., taking on multiple jobs, avoiding triggers) • Concealing distress in social/professional domains

### Second round of delphi study

Of the 18 experts who participated in the first round, 16 completed the second round. These experts provided importance ratings for each of the thematic dimensions supporting optimal functioning among individuals with mental illness. Following this, the mean scores were calculated, and group ranks were assigned. Lower mean rank values indicate higher perceived priority. As shown in Table 3, MPS emerged as the highest-priority dimension (mean rank = 2.63), followed by SFS (3.06) and PHW (3.69). Mid-range rankings were assigned to ECC (4.19), MSP (4.50), and SDL (4.81). SDSA (5.50) ranked seventh, while FAM received the highest mean rank (7.63), indicating that experts considered it the least desirable or least central pathway for sustaining optimal functioning.

Overall, the ranking pattern suggests a clear expert consensus that formal clinical treatment, supportive interpersonal environments, and physical health practices are foundational to optimal functioning. In contrast, compensatory or masking-based strategies were viewed as comparatively less beneficial and more peripheral to sustained recovery.

**Table 3:** Second-round delphi feedback

Expert	MPS	SDL	MSP	PHW	SFS	ECC	SDSA	FAM
E1	4	6	7	5	2	3	1	8
E2	1	7	2	3	5	4	6	8
E3	8	1	2	6	3	4	5	7
E4	1	3	4	6	2	5	7	8
E5	3	2	6	1	5	4	7	8
E6	1	6	5	4	2	3	8	7
E7	1	8	3	6	2	4	5	7
E8	8	7	5	3	1	2	4	6
E9	1	6	2	3	4	5	7	8
E10	1	6	7	5	2	4	3	8
E11	1	6	5	3	2	4	7	8
E12	1	4	8	2	3	6	5	7
E13	1	3	4	2	6	5	7	8
E14	5	7	6	2	4	1	3	8
E15	3	4	2	5	1	6	7	8
E16	2	1	4	3	5	7	6	8
Mean	2.63	4.81	4.50	3.69	3.06	4.19	5.50	7.63
Group Rank	1	6	5	3	2	4	7	8

Note: Medical and Professional Support, MPS; Structured Daily Living, SDL; Mindfulness and Spiritual Practice, MSP; Physical Health and Wellness, PHW; Social and Family Support, SFS; Emotional and Cognitive Coping, ECC; Self-Directed Support and Autonomy, SDSA; Functional Adaptation and Masking, FAM

Agreement among experts’ rankings was assessed using Kendall’s coefficient of concordance. For the 16 experts rating eight dimensions, the analysis yielded Kendall’s W = 0.41, χ²(7) = 45.88, *p* <.001. In Delphi research, W values above 0.40 are typically interpreted as indicating moderate consensus [17]. Accordingly, the obtained W value of 0.41 reflects a statistically significant but relatively modest level of agreement. This degree of concordance is not unexpected given the heterogeneity of the experts, who comprised practitioners and researchers from varied professional backgrounds and service settings who may reasonably differ in how they prioritise aspects of functional support.

Nevertheless, the highly significant p-value, the stability of the overall ranking structure, and the study’s exploratory aim to establish a preliminary prioritisation rather than a definitive hierarchy indicate that this level of agreement was sufficient. Therefore, the second-round results were accepted as the final consensus ranking, and no additional Delphi rounds were conducted.

## Discussion

This study set out to identify and prioritise the strategies that most meaningfully support optimal functioning among people living with mental illness. The eight dimensions that emerged, together with their expert-derived ranking, do more than summarise helpful practices. They illuminate how functioning is displayed through multiple pathways, namely the interaction among clinical care, daily routines, social ecologies, and often invisible forms of adaptation. Read alongside the Recovery Model, the Biopsychosocial Model, and behavioural self-management frameworks, the findings offer a layered account of functional recovery that speaks directly to the structural gap highlighted in the introduction. This multidimensional perspective is built on interconnected biological, psychosocial, and behavioural factors that intersect across multiple levels. These layers present a complex but more complete picture than a single-lens perspective could provide.

### Recentering functioning within recovery frameworks

Experts placed Medical and Professional Support, Social and Family Support, and Physical Health and Wellness at the top of the hierarchy. This pattern reinforces the central idea of contemporary recovery models that functional gains are unlikely to be sustained without a foundation of clinical stability and environmental support [31, 47]. The experts’ descriptions were not abstract. One psychiatrist emphasised the need for “*optimisation of medicine*,* psychotherapies*,* relapse prevention strategies*,* rehabilitation programs such as ADL training… and supported employment*” (E3), while another observed that many individuals “*attend therapy with professional help and take medication*” (E4). These accounts echo work on integrated and recovery-oriented care, where clinical treatment, psychosocial rehabilitation, and vocational support are conceived as mutually reinforcing components rather than sequential phases of recovery [18, 54]. Similarly, Cook and Chambers’ [21] study reported that while personal strengths were important, support, therapy and practical help from mental health staff enabled people to develop these personal coping strategies.

The prominence of Social and Family Support further demonstrates the relational core of recovery and aligns closely with the biopsychosocial model. Experts repeatedly described individuals *“living with their family or significant others to gain optimal support*,*” “joining a support group*,*”* and *“seeking social support”* (E5; E18). Others highlighted everyday relational coping strategies such as *“talking to someone else… going for a walk… distraction… or changing the environment”* (E8). These observations are consistent with relational and ecological models of functioning, which conceptualise recovery as emerging from ongoing exchanges between individuals and their social worlds, rather than as an exclusively internal or clinical process [48]. Within this lens, the experts’ ranking can be interpreted as a call to design systems that position family and community networks as core resources in recovery integral, not optional, components of sustainable mental health care [1].

Physical Health and Wellness, in third place, aligns with the biopsychosocial model’s emphasis on biological and lifestyle factors that influence mental health. Experts linked physical activity, sleep, and diet to mental health in concrete, functional terms. One expert summarised common practices as *“physical activity*,* mindfulness*,* dietary modification*,* social connection and sleep hygiene”* (E7), while another noted that individuals commit to *“regular exercise which in turn also helps one’s mental health”* (E2). These insights align with evidence demonstrating that physical health practices, self-care, and biological stability form the basic infrastructure of psychological and functional recovery rather than being peripheral to it [6]. Across recovery literature, recent evidence converges on the view that functioning must be recentred within recovery frameworks. Sustained recovery depends on the interaction of clinical stability, relational support, and physical wellness, a triad that defines recovery not merely as symptom reduction, but as the restoration of meaningful, socially embedded functioning [1, 9, 47].

### Everyday regulation and the work of self-management

The mid-ranked dimensions of Emotional and Cognitive Coping, Mindfulness and Spiritual Practice, and Structured Daily Living capture the ongoing work individuals undertake to translate clinical stability into everyday functioning. These domains align with behavioural self-management frameworks, which emphasise self-regulation, goal setting, and habit formation as core processes linking symptom control with adaptive functioning [80, 81].

Experts described how individuals use combinations of distraction, goal setting, and acceptance to manage emotional demands. One expert noted the pattern of *“distraction (getting several jobs and always staying busy*,* scrolling the phone*,* playing games)*,* acceptance*,* setting goals”* (E8). Another emphasised *gratitude practice* as a means of perspective-shifting (E16), while a third highlighted *self-motivation and emotional awareness* (E17). These descriptions mirror growing empirical evidence that flexible, multi-strategy coping repertoires integrating emotion regulation, cognitive reframing, and behavioural activation support resilience and adaptation across changing contexts [3, 97]. Theoretically, these findings align with models of self-regulatory flexibility, which propose that adaptive coping depends on the ability to switch between cognitive, emotional, and behavioural strategies to maintain goal pursuit under stress [82].

Structured Daily Living brings these processes into the material organisation of everyday life. Experts described individuals using *“a good morning schedule… journaling… night routine to unwind negative thoughts… [and] using available resources online”* (E2). Within behavioural self-management models, such routines serve as environmental cues and habit scaffolds, reducing cognitive load and sustaining engagement in valued activities [101]. Recent evidence supports this view that digital self-regulation and app-based psychoeducational tools enhance emotion regulation and adaptive coping in everyday settings by embedding behaviour change in structured routines [67]. The mid-ranking of this dimension suggests that experts see routines as necessary stabilisers of functioning, but most effective when built on the higher-ranked foundations of treatment, social support, and physical health.

Mindfulness and Spiritual Practice sit at the intersection of personal recovery and functional adaptation. Experts pointed to *“mindfulness therapy”* and *“meditation”* (E11), as well as *“journaling*,* meditation*,* self-care exercises*,* spiritual beliefs*,* self-motivation”* (E17). One explicitly linked gratitude to cognitive balance, describing it as a way to *“focus on things that are positive in life to counterbalance negative thinking”* (E16). These insights align with recent research demonstrating that mindfulness training enhances emotional self-regulation, cognitive reappraisal, and stress resilience while reducing suppression and rumination [27, 36]. These mechanisms bridge the personal recovery emphasis on meaning, hope, and acceptance with the behavioural emphasis on sustained goal-directed activity and adaptive emotion regulation. Across recent studies, everyday regulation emerges as the practical core of self-management and recovery. Emotional and cognitive coping, mindfulness, and daily structure represent interlocking mechanisms that translate clinical improvement into durable functioning by cultivating self-regulation, habit stability, and meaning-making [67, 97]. This is supported by Cook and Chambers’ [21] findings, which showed that engaging in specific activities in one’s daily life also helped with mental health, and vice versa. The benefits of an individual approach contribute to the development of self-help and self-monitoring strategies [49].

### Autonomy, invisible labour, and the limits of individual responsibility

The lower ranking of Self-Directed Support and Autonomy should not be interpreted as suggesting that autonomy is unimportant. Experts offered numerous examples of individuals taking initiative, *“reaching for self-help materials first*,* then asking for help from others”* (E4), or relying on *“self-help resources*,* online tools*,* educational materials*,* and self-motivation”* (E17). Hitch et al., [49] reported that the experience of engaging in activities of daily living is strongly related to a sense of self and identity; achievements derived from these activities became part of many clients’ coping and survival strategies. Within self-management theory, these behaviours exemplify self-efficacy and an internal locus of control, both of which are associated with better outcomes and recovery trajectories [61]. From a Self-Determination Theory perspective, autonomy is an essential psychological need that promotes intrinsic motivation, competence, and well-being, yet it is also dependent on supportive environments that nurture rather than demand self-governance [78].

However, the collective decision to place autonomy below more structural supports signals a deeper insight. In under-resourced or fragmented systems, exhortations to self-manage can shift responsibility onto individuals without providing the social, clinical, or material scaffolding needed for success [73]. The relational autonomy framework challenges such individualistic models by recognising that autonomy is socially embedded and interdependent, shaped by relational, economic, and institutional contexts rather than exercised in isolation. Within the biopsychosocial model, this critique underscores that overemphasising personal agency neglects systemic barriers, such as poverty, stigma, and service fragmentation, that determine what forms of “self-management” are actually feasible. Thus, autonomy is most constructive when scaffolded by accessible care, social connection, and supportive physical environments [41, 64].

The final dimension, Functional Adaptation and Masking, deepens this critique. Experts described individuals who *“try to overcompensate and sometimes show apparent competence… sometimes change activity in order to attain optimal functioning”* (E1) and others who survive by *“distraction*,* getting several jobs and always staying busy”* (E8; E15). These strategies mirror what the literature calls *apparent competence*, or *masking*, in which outward functionality is maintained at a high internal cost. Such compensatory strategies reflect the invisible labour of coping with the continuous, energy-intensive effort to perform normality within unsupportive environments [34]. Theoretically, these can be read as emergency adaptations, temporary alignments between personal survival and external expectations rather than sustainable forms of recovery.

The low ranking of this dimension is therefore not a dismissal of its prevalence but an ethical stance. It reflects recognition that mental health systems should neither valorise nor rely on functioning sustained through overwork, concealment, and self-erasure. In relation to the Recovery Model, this distinction marks a boundary between visible role performance and genuine personal recovery, which depends on integration, self-acceptance, and supported autonomy rather than compensatory effort [26]. From a biopsychosocial perspective, it reveals how institutional pressures and social norms can distort adaptive processes into burdensome survival strategies that demand “resilience” when structural reform is required. In summary, research supports a relational and contextual understanding of autonomy, challenging the over-individualisation of recovery. Autonomy contributes to well-being and motivation only when embedded in supportive systems that enable rather than demand self-management [61, 73]. When autonomy becomes detached from relational and structural supports, it risks transforming into invisible labour, the hidden work of sustaining functionality under constraint rather than flourishing within freedom.

The discussion illustrates that participants’ reports align closely with recovery-oriented models, such as the Integrated Recovery Oriented Model proposed by Frost et al., [31], which posits that a broad range of both non-professional and professional recovery contexts can contribute to functioning [31]. These contexts may include small, self-initiated steps, such as engaging in everyday activities that foster independent living and autonomy, as well as structured support received from family members and mental health services ([9, 42]. The findings of this study extend this framework by integrating expert-derived insights with evidence from existing qualitative research on lived experiences, thereby situating the results within a broader empirical context rather than relying solely on direct primary accounts. This approach is consistent with calls for integrated, multilevel recovery implementation [10]. The reports from mental health professionals in this study align with the experiences of individuals with a diagnosed mental illness reported in previous studies (e.g., Cook and Chambers, [21], Hitch, [49], Hovland, [50]. These studies provide contextual support for the present findings, but are not primary data sources in this Delphi study. Among other findings on self-regulation and social and familial engagement, one important insight regarding individual choices stands out. These reports illustrate the importance of individual choices in developing coping strategies that individuals value for their mental health. The achievements stemming from these choices contribute to self-concept and identity, which in turn reinforce personal initiative. Therefore, families and healthcare professionals should encourage individuals to initiate and pursue activities that they consider meaningful for maintaining their mental health. Finally, the recognition that recovery is highly personal, self-directed, and achievable through multiple pathways is strongly supported in the literature [33].

### Recommendations for mental health professionals

The findings underscore the multidimensional nature of functional recovery and highlight the critical role mental health professionals can play in shaping the environments, relationships, and therapeutic frameworks that enable individuals to sustain daily functioning. Contemporary scholarship has increasingly argued that clinical practice must move beyond narrow biomedical interpretations and instead embrace integrated, recovery-oriented approaches that recognise functioning as an emergent property of clinical stability, social connection, autonomy, and environmental support [23, 55]. In this sense, mental health professionals become key architects of the conditions under which effective strategies for optimal functioning can be enacted.

A central implication concerns the need to conceptualise functioning as a primary therapeutic outcome. Clinical encounters should be structured around coordinated, integrated care pathways involving psychiatric treatment, skills-based rehabilitation, psychosocial support, and vocational engagement. Evidence shows that integrated recovery models, where treatment and community participation are advanced simultaneously, produce more sustainable improvements in daily functioning and quality of life [31, 63]. Mental health professionals should therefore collaborate closely with rehabilitation teams, peer support workers, and community organisations to ensure that functional goals are embedded in routine care rather than treated as secondary or optional.

Given the strong influence of interpersonal environments on functioning, practitioners should actively position family members, significant others, and community networks as core therapeutic resources. Systematic reviews highlight that family involvement remains significantly underutilised despite its demonstrated benefits for sustained recovery and stability [66]. Research from European and Asian contexts further emphasises that recovery is inherently relational and context-dependent, shaped by social embeddedness and environmental meaning-making rather than solely by individual resilience [56, 91]. Mental health professionals can leverage these insights by facilitating psychoeducation, building collaborative alliances with families, and supporting community participation as part of therapeutic planning.

Professionals should also integrate self-regulatory and behavioural strategies directly into therapeutic processes. Functional recovery relies heavily on individuals’ capacities to regulate emotions, structure routines, and cultivate adaptive habits. Interventions such as behavioural activation, goal setting, mindfulness practices, and psychoeducation have been shown to enhance both symptom control and daily role functioning [72]. Incorporating digital tools and self-monitoring methods can further support individuals in maintaining these strategies beyond therapeutic sessions, helping them translate clinical gains into everyday stability.

Another essential consideration is fostering autonomy in ways that are supportive rather than burdensome. While personal agency is fundamental to psychological well-being, research cautions against models that overemphasise individual responsibility in contexts where systemic supports are insufficient [71, 75]. Mental health professionals should adopt shared decision-making frameworks, help patients articulate meaningful goals, and ensure that autonomy is exercised within relationally supportive and resource-rich environments. This approach aligns with recovery-oriented practice, which positions autonomy not as independence from others but as the ability to act purposefully within supportive networks.

Professionals must also remain attentive to high-cost forms of functioning, such as masking, overwork, or emotional suppression, which may outwardly appear adaptive but internally place individuals at risk. Scholars warn that such strategies often reflect attempts to meet social or occupational expectations while concealing distress, and they may contribute to burnout or relapse [55]. Person-centred recovery frameworks emphasise the importance of distinguishing outward performance from genuine well-being, advocating for clinical spaces where individuals can explore and replace burdensome coping strategies with sustainable alternatives [57].

Context-sensitive, personalised intervention planning remains essential. Functional strategies cannot be universally prescribed; they must be tailored to the individual’s cultural background, social conditions, coping patterns, and environmental constraints [63]. Such personalised approaches enhance treatment adherence, equity, and long-term sustainability, consistent with the biopsychosocial understanding of mental health [23]. Digital approaches can be used efficiently to deliver personalised prompts that reinforce the skills introduced through cognitive and behavioural approaches. In this way, an integrated system based on psychosocial, community-based and digital approaches can be developed to support individuals.

Finally, mental health professionals should engage in interdisciplinary collaboration to address structural determinants that shape functioning. Housing instability, employment difficulties, and fragmented support systems represent substantial barriers to daily functioning, and evidence suggests that integrated service models across mental health, social care, and municipal systems produce more holistic recovery outcomes [55]. Clinicians also play important advocacy roles by identifying systemic gaps and promoting policies that support social equity and justice within mental health care [57].

These recommendations reflect a shift toward multidimensional, relational, and system-aware professional practice. Mental health professionals are not merely providers of treatment; they shape therapeutic ecosystems that influence how individuals live, function, and participate in daily life. By adopting integrated care, strengthening social and familial engagement, reinforcing behavioural self-management, supporting autonomy, addressing maladaptive functioning, personalising interventions, and collaborating across sectors, professionals create the conditions for sustained functional recovery [55, 56, 63].

## Conclusion

This study addressed a persistent structural problem in mental health care, namely the misalignment between systems organised around symptom containment and the lived reality of people whose long-term well-being depends on sustained functioning in everyday life. Despite decades of progress in clinical knowledge and an expanding repertoire of psychosocial, community, and digital interventions, support for functioning remains fragmented and uneven. In response to this gap, the present study used a modified Delphi design to consolidate expert judgment into a theory-informed and empirically grounded framework that identifies and prioritises strategies for optimal functioning among individuals living with mental illness.

Across two rounds, experts generated 40 strategy statements, which were synthesised into eight dimensions. The final ranking pattern highlighted three domains as foundational anchors of functional recovery, namely formal clinical care, supportive interpersonal environments, and physical health practices. Mid-ranked domains captured everyday regulation and habit formation. In contrast, the lowest-ranked domains highlighted the limits of individual responsibility in under-resourced systems and the high personal cost of masking and overcompensation. The findings demonstrate that functioning is constructed at the intersection of treatment access, social ecology, lifestyle stability, self-regulation, and often invisible labour.

The study carries several key implications for practice, systems, and theory. For frontline professionals, the findings support a shift in orientation in which functioning becomes a primary therapeutic target rather than a secondary outcome that is assumed to follow symptom reduction. Integrated care pathways that combine psychiatric treatment, psychosocial and vocational rehabilitation, physical health promotion, and community participation appear especially important for sustainable recovery. The high ranking of Social and Family Support underscores the relational character of functioning and argues for more systematic involvement of families, peers, and community networks in assessment, planning, and intervention. The mid-ranked domains suggest that clinicians should deliberately cultivate emotional regulation, coping flexibility, mindfulness practices, and structured routines as mechanisms that carry clinical gains into everyday settings. At the same time, the relatively lower ranking of autonomous self-help and high-effort adaptation can be read as a caution against policy and service models that quietly shift responsibility for functioning onto individuals while leaving structural barriers intact. Conceptually, the framework strengthens relational and contextual readings of the Recovery Model, the Biopsychosocial Model, and behavioural self-management approaches, showing how these perspectives are translated into concrete strategic priorities in real-world practice.

These contributions should be interpreted in light of several limitations. The expert, although professionally diverse, was modest in size and situated within specific service and cultural contexts, which may constrain the transferability of the ranking structure to other regions or systems. The consensus reflects professional viewpoints rather than co-produced perspectives that directly include people with lived experience and their families. The level of agreement, while statistically significant, was moderate, consistent with the expert’s heterogeneity but indicating that reasonable differences in emphasis remain. In addition, the thematic organisation relied on inductive analysis supported by AI-assisted tools and subsequent human review, an approach that, while transparent and reflexive, still involves interpretive judgement and could yield somewhat different structures if replicated by other teams.

These limitations point toward clear directions for future work. Subsequent studies could co-design and refine the framework with service users, carers, and peer workers, ensuring that lived experience is embedded alongside professional insight. Additionally, future work could be expanded to include other participants who work in community mental health settings, such as social workers, nurses, and occupational therapists. Quantitative and mixed-methods research could test the relative contribution of each dimension to functional outcomes across diagnoses, cultures, and service configurations, including longitudinal designs that track functioning across transitions such as discharge, return to work, or changes in housing. Implementation research is needed to explore how services can embed the higher-ranked dimensions into everyday practice and how organisational and policy levers can address structural barriers related to housing, employment, and social protection. Digital and hybrid interventions could be explicitly designed around this framework to clarify how technology can support, rather than displace, relational and environmental forms of care.

In conclusion, the study goes beyond the general assertion that functioning is important and offers a structured, consensus-based map of the strategies that experts consider most critical for helping people with mental illness live well in their daily lives. The findings foreground a triad of clinical stability, relational support, and physical wellness, supported by everyday self-regulatory practices and bounded by ethical concerns about over-individualised responsibility and high-cost masking. This framework provides a practical scaffold for clinicians, service leaders, and policymakers seeking to reorient mental health systems toward cultivating human capacities, agency, and participation. It invites a reframing of the central question from whether individuals are resilient enough to function within current conditions to whether systems are willing and able to create the social, clinical, and material conditions under which genuine functioning and recovery can be realised.

## Data Availability

The datasets generated and analysed during the current study consist of anonymised expert responses. These data are available from the corresponding author upon reasonable request. Due to confidentiality protections associated with the Delphi process, full raw qualitative responses cannot be made publicly available.
